# A comparative parasitological, histopathological, and proteomic analysis of *Schistosoma mansoni* infected mice treated with ivermectin and praziquantel

**DOI:** 10.3389/fvets.2025.1646155

**Published:** 2025-07-29

**Authors:** Eman Sayed El-Wakil, Mona Mohamed Tolba, Mona Hasan El-Sayad, Maha Khairy Hassen, Hayat S. Al-Rashidi, Mina A. Almayouf, Wafa Abdullah I. Al-Megrin, Hind Alzaylaee, Esraa Abdelhamid Moneer

**Affiliations:** ^1^Department of Parasitology, Theodor Bilharz Research Institute, Kornaish El-Nile, Giza, Egypt; ^2^Parasitology Department, Medical Research Institute, Alexandria University, Alexandria, Egypt; ^3^Department of Medical Laboratory Technology, Faculty of Applied Health Sciences Technology, Pharos University in Alexandria, Alexandria, Egypt; ^4^Department of Biology, College of Science, Qassim University, Buraydah, Saudi Arabia; ^5^Department of Biology, College of Science, Princess Nourah bint Abdulrahman University, Riyadh, Saudi Arabia

**Keywords:** ivermectin, praziquantel, proteomic analysis, scanning electron microscope, *Schistosoma mansoni*

## Abstract

This study was designed to compare the effectiveness of ivermectin (IVM) with praziquantel (PZQ) in treating *Schistosoma mansoni*-infected mice through biological and proteomic analysis. Detecting protein structure changes in the worms after treatment could help pursue drug efficacy in schistosomiasis. Sixty Swiss albino mice were infected with *S. mansoni* cercariae and were divided into three major groups (Infected untreated control, praziquantel-treated, and ivermectin-treated). The evaluation of treatment was performed by parasitological, histopathological analysis, scanning electron microscopy (SEM), and proteomic analysis of the worms through lysis and protein extraction, SDS-PAGE, Mass Spectrometry, and data analysis. The treated groups significantly reduced mean worm load and ova count with smaller granulomas compared to the infected control group. In adult worms treated with PZQ and IVM, severe tegumental destruction, peeling, erosion, ulceration, and suckers damage were detected by SEM. The proteomic study identified 19 protein bands, 12 commonly shared proteins between all studied groups, and seven differential protein bands. Molecular and biological function administered from the NCBInr database revealed the presence of glycolytic proteins, structural proteins, and cytosol stress response. Although praziquantel outperformed ivermectin, the anti-schistosomal properties of ivermectin are encouraging, evidenced by changes in the protein structure of the worms detected after ivermectin treatment. This may open the way to use ivermectin in combination with other anti-schistosomal medicines to avoid any potential resistance from monotherapy. Besides, it highlights the role of proteomic analysis in differential protein identification that could help efficiently treat schistosomiasis.

## Introduction

In tropical and subtropical areas, schistosomiasis is a widespread parasitic disease that substantially threatens human health and impedes social and economic development (1). Schistosomiasis, the second most prevalent parasitic disease in the world after malaria, is a major contributor to morbidity. The disease is thought to impact around 251.4 million people worldwide in 78 different countries, and it is the cause of about 280,000 fatalities annually (2, 3). The three major schistosomes infecting humans are *Schistosoma* (*S*.) *mansoni, S. japonicum*, and *S. haematobium* (4). *S. mansoni* eggs cause granuloma formation, inflammation, fibrosis, and damage to the liver, which can result in portal hypertension and hinder daily activities (5).

Praziquantel (PZQ) is the preferred option for treating schistosomiasis (6, 7). PZQ is well tolerated and effective generally against adult worms of all schistosome species, with only minor and temporary adverse effects (8). Meanwhile, juvenile schistosomula stages can retain viability after PZQ chemotherapy and progress to adulthood. The appearance of lower PZQ efficacy as a result of mass drug administration (MDA) aimed at people in tropical and subtropical areas where schistosomiasis is endemic foreshadows the emergence of drug-resistant variants. In addition, PZQ shows no protection against re-infection, leading to complex control strategies in high-endemic areas. Furthermore, no vaccination or medication prevents infection, which adds to the disease burden (7).

Ivermectin (IVM) is an antiparasitic medication that the Food and Drug Administration has licensed. This macrocyclic lactone also possesses antibacterial and antiviral activities. It treats various helminth infections with high efficacy and safety. In addition to being the first line of treatment for onchocerciasis, strongyloidiasis, and scabies, it is effective against other soil-transmitted helminths. It is also a component of the lymphatic filariasis regimen (9, 10). IVM's possible ability to prevent schistosomiasis has been examined in previous researches; however, its effectiveness varies according to the *Schistosoma* species, life stage, and treatment regimen. For instance, IVM significantly reduced worm counts and intestinal and hepatic tissue egg load in *S. mansoni* experimentally infected mice (11). Nonetheless, another study questioned ivermectin's efficacy in treating mice infected with *S. mansoni* (12). IVM targets the schistosome-expressed glutamate-gated chloride channel, leading to parasite paralysis and death (9, 13). A relatively low affinity of the *S. mansoni* glutamate-gated chloride channel for IVM may be the reason for the inconclusive evidence base regarding the drug's effectiveness against *S. mansoni* (14). Consequently, additional research is required to validate IVM's effectiveness in managing schistosomiasis.

When comparing the effectiveness of PZQ and IVM on the development stages in the life cycle of schistosomes, it was reported that PZQ exhibits little activity against schistosomula, juvenile worms, and eggs. However, it is efficient against adult schistosomes and cercariae (7). On the other hand, IVM may affect several stages of the life cycle, albeit with varying degrees of effectiveness (15). IVM at a dose of 0.2 μg mL^−1^ was adequate to kill 50% of the cercariae and 90% of the miracidia within 5 and 30 min, respectively (15).

Drug combinations may offer an alternate approach to treating diseases, given the benefits of improved efficacy, reduced toxicity, and decreased chance of drug resistance in chemotherapy (16). The potential advantages of combining IVM with PZQ have been investigated. In Mass drug administration (MDA) programs, the co-administration of PZQ, IVM, and albendazole indicated that such combinations are generally safe (17). Additionally, an IVM/PZQ suspension was developed by Tang et al. (18) with documented improvement of pharmacokinetics in the combination product.

Proteins are essential for a variety of biological functions. The dynamic processes of life are rooted in their composition and interactions. The term “proteomic analysis” describes the methodical identification and measurement of a biological system's (cell, tissue, organ, biological fluid, or organism) entire protein complement (the proteome) at a particular moment in time (19). Three fundamental technological pillars constitute the basis of proteomics: a technique for fractionating complex protein or peptide mixtures; mass spectrometry (MS) for gathering the information required to identify individual proteins; and bioinformatics for analyzing and compiling the MS data (20).

Researchers have recently gathered a significant amount of knowledge about human physiological and pathological processes by studying biomarkers to identify diseases and determine the principles of pathological abnormalities (20) due to the development of proteomics technology (21, 22). Using cutting-edge mass spectrometric technology and phosphoprotein enrichment techniques, the mechanisms of action of numerous medications were successfully unraveled (23). Understanding biological processes at the protein level is now made possible by MS-based proteomics. Over the past 15 years, significant advancements have been made in proteomics with high accuracy and sensitivity (24).

This study was designed to compare the effectiveness of ivermectin (IVM) with praziquantel (PZQ) in treating *Schistosoma mansoni*-infected mice through biological, ultrastructural, and proteomic analysis. Additionally, detecting protein structure changes in the worms pre- and post-treatment using proteomic methods could help pursue drug efficacy on schistosomiasis. This could improve our knowledge about how the drugs used in schistosomiasis treatment work at the molecular level.

## Methods

### Experimental animals and design

Theodor Bilharz Research Institute (TBRI), Giza, Egypt, provided the 60 inbred Swiss Albino male mice (4–6 weeks old, 20–25g) used in this study, and marked as parasite-free. The mice were isolated in separate cages and fed a regular diet and water. Following approval from the Institutional Animal Care and Use Committee of Alexandria University (ALEXU-IACUC) with approval NO. (Au0122272221), all methods were performed per the internationally recognized regulations and adhered to the ARRIVE guidelines.

Twenty mice were placed in each of the three primary groups, which were distributed evenly among the mice:

Group I: untreated infected mice,
Group II: infected and treated with PZQ [Epiquantel tablets (600 mg) from Egyptian International Pharmaceutical Industries Company (EIPICO)] at a dose of 500 mg/kg/body weight (bwt) for two successive days through oral gavage at 42 dpi (25) and
Group III: infected and treated with IVM [Iverzine tablets (6 mg) from Universal Industries Pharmaceuticals Corporation (PHARMA)] at a dose of 1mg/kg for three consecutive days through oral gavage at 42 days post-infection (dpi) (9).

### *Schistosoma mansoni* infection

*Biomphalaria alexandrina* snails infected with *S. mansoni* miracidia were provided by the TBRI *Schistosoma* Biological Supply unit. Mice used in experiments were infected with *S. mansoni* cercariae that were shed and harvested from infected snails. Each mouse was subcutaneously injected with 60 ± 10 *S. mansoni* cercariae, which was considered 0 dpi (26).

### Parasitological examination, including recovery of the worms, oogram pattern, and count of the tissue eggs

At the end of the experiment (60 dpi), the mice were euthanized under light anesthesia by isoflurane inhalation. The hepato-portomesentric perfusion technique was applied to sacrificed mice to collect adult *S. mansoni*, quantify the worm load, identify sex (male, female, or copula), and then calculate the percentage of worm reduction (27). The obtained worms were then divided into two parts, one for scanning electron microscope (SEM) examination and the other for proteomic analysis.

For oogram studies, 100 eggs per oogram were randomly selected from the small intestine and sorted into three categories: immature, mature, and dead. For each type, the percentage was then calculated (28). Additionally, the liver and intestine (one gram) were incubated at 37°C in 4% KOH for 18 h for tissue egg count to determine the number of eggs per gram of tissue (29).

### Histopathological studies

For the histopathological analysis, utilizing Masson's Trichrome (MT) specific stain and Hematoxylin and Eosin (H&E) as routine stains, dissected liver specimens were fixed using 10% formalin according to the reported procedures (30).

### Scanning electron microscope examination (SEM)

Worms obtained from the three study groups were washed in phosphate-buffered saline (pH 7.4), treated in 2.5% glutaraldehyde for 24 h at 4°C, and processed for SEM inspection utilizing Jeol-JSM-820 at the Electron Microscopy Unit of the Faculty of Science, Alexandria University (31).

### Preparation of protein extracts

*S. mansoni* protein extracts were obtained using the Trizol protocol (Thermo Fisher Scientific), according to the manufacturer's instructions (Thermo Fisher Scientific Invitrogen User Guide). Briefly, the *S. mansoni* worms were lysed and homogenized directly in Trizol reagent at room temperature (=25°C) (24). The homogenized samples were incubated at room temperature to permit complete dissociation of the nucleoprotein complex. After homogenization, we proceeded to the separation phase, adding chloroform and centrifuging the samples. The aqueous phase was removed, and the interphase and organic phenol-chloroform phase were used for protein isolation. Next, isopropyl alcohol precipitation was performed, and the pellet was solubilized in SBI buffer (7 mol/L urea, 2 mol/L thiourea, 0.015 mol/L DHPC, 0.5% Triton X-100, 0.02 mol/L DTT, and complete mini protease inhibitor cocktail tablets) and stored at −80°C until use (32). Protein concentration in protein extracts was measured by Bradford assay, and the quality of the extract was verified in 12% uniform SDS-PAGE gels (33).

### SDS-PAGE gel electrophoresis

SDS-PAGE gel electrophoresis was performed in triplicate with 240 μg of protein extracts. To prepare samples for SDS-PAGE, protein samples were diluted in a rehydration solution containing 7 mol/L urea, 2 mol/L thiourea, 4% CHAPS, 0.5% IPG buffer, 1% DTT, and 0.002% bromophenol blue. The rehydration was carried out passively overnight for 12 h in a 13 cm pH 3–10 strip. The strips were then applied on an Ettan IPGphor 3 System for protein separation by isoelectric focusing (IEF), using the following conditions developed for this work: at a constant current of 50 μA/strip, the voltage program started with a gradient up to 3.5 kV in 3 h, then a step of 3 h at 3.5 kV, then a total voltage of 64.0 kVh to the end. After focusing, the strips containing proteins were reduced in an equilibration solution (50 mol/L Tris–HCl, pH 8.8, 6 mol/L urea, 20% glycerol, 2% SDS) containing 2% DTT, and then alkylated in the same solution containing 2.5% IAA. The Immobilized pH gradient (IPG) strips and molecular weight standards were then transferred to the top of 12% uniform SDS-PAGE gels and sealed with 0.5% agarose (34). Data was analyzed using Total Lab analysis software, https://www.totallab.com, and the Gel Documentation System (GelDoc-it, UVP, England, Ver. 1.0.1).

### Mass spectrometry (MS)

Mass spectrometry analysis was used to identify and quantify the protein extract from the adult worms extracted from the control group (group I) and the treated groups (II, III). The SDS-PAGE gel's protein bands were manually and separately removed, and then trypsin was used for digestion. Acclaim PepMap 100 C18 trap and Acclaim PepMap RSLC C18 column (Thermo Fisher Scientific, USA) (Exigent, Canada) were used to separate the peptides using a nano-LC Ultra 2D plus loading pump and a nano-LC as-2 auto-sampler. The peptides were eluted according to (35).

### Mass spectrometry data analysis

The UniProt and Scientific Data Sharing Information Bio information protein databases for *Schistosoma spp*. were used for all searches (https://www.uniprot.org). Using data from the Gene Ontology database, the detected proteins' molecular function and biological process were assigned (36).

### Statistical analysis

The experimental groups were statistically compared using SPSS software version 22. There were four types of descriptive statistics: counts, percentages, arithmetic mean, and standard deviation. The following formula was used to calculate the percentage of reduction between the treated and untreated groups: (mean value of untreated group- mean value of treated group) x 100/mean value of untreated group. All statistical tests were conducted at a 5% significance level (at 95 % confidence interval). To compare the means of two corresponding groups, a one-way analysis of variance (ANOVA) was used, followed by an independent two-sample *t*-test. *P-values* higher than 0.05 were considered statistically insignificant.

## Results

### Parasitological results

A comparison of worm burdens and ova developmental stages among the groups under study is shown in Tables 1, 2. Regarding copula and male worm burdens, they demonstrated a statistically significant decrease in the mean worm load harvested from groups II (PZQ) and III (IVM) compared to the infected control. At the same time, mean female worm counts did not differ statistically between PZQ and IVM compared to the control. Compared to the control group (20.80 ± 2.8), group II showed a statistically significant reduction in mean total worm loads (2.40 ± 1.58), followed by group III at 13.0 ± 2.54. Groups II and III ova of treated adult worms were smaller and more spherical than those from control worms (group I).

**Table 1:** Comparison between worm burdens among the studied groups.

**Worm burdens**	**Group I (*n* = 20)**	**Group II (*n* = 20)**	**Group III (*n* = 20)**	***F***	***P***
**No. Copula**					
Min. – Max.	7.0–11.0	0.0–2.0	3.0–7.0	145.576^*^	<0.001^*^
Mean ± SD	8.90 ± 1.20	0.70 ± 0.67	5.40 ± 1.26		
Median (IQR)	9.0 (8.0–10.0)	1.0 (0.0–1.0)	5.50 (5.0–6.0)		
**Sig. between groups** *p*_1_ < 0.001^*^, *p*_2_ < 0.001^*^, *p*_3_ < 0.001^*^					
**No. Males**					
Min. – Max.	1.0–3.0	0.0–2.0	0.0–2.0	10.969^*^	<0.001^*^
Mean ± SD	2.20 ± 0.79	0.80 ± 0.63	1.20 ± 0.63		
Median (IQR)	2.0 (2.0–3.0)	1.0 (0.0–1.0)	1.0 (1.0–2.0)		
**Sig. between groups** *p*_1_ < 0.001^*^, *p*_2_ = 0.008^*^, *p*_3_ = 0.408					
**No. Females**					
Min. – Max.	0.0–2.0	0.0–1.0	0.0–1.0	2.625	0.091
Mean ± SD	0.80 ± 0.79	0.20 ± 0.42	0.40 ± 0.52
Median (IQR)	1.0 (0.0–1.0)	0.0 (0.0–0.0)	0.0 (0.0–1.0)
**Sig. between groups** ***p*_1_ < 0.05, *p*_2_ < 0.05, *p*_3_ < 0.05**					
**No. Total**					
Min. – Max.	17.0–25.0	0.0–5.0	8.0–16.0	151.508^*^	<0.001^*^
Mean ± SD	20.80 ± 2.82	2.40 ± 1.58	13.0 ± 2.54		
Median (IQR)	20.5 (18.0–23.0)	2.0 (1.0–4.0)	12.5 (12.0–15.0)		
**Sig. between groups** *p*_1_ < 0.001^*^, *p*_2_ < 0.001^*^, *p*_3_ < 0.001^*^					

IQR, Interquartile range; SD, Standard deviation.

F, F for ANOVA test, pairwise comparison between the study groups was done using the *Post Hoc* Test (Tukey).

*p*: *p*-value for comparing the studied groups.

*p*_1_: *p*-value for comparing group I and group II.

*p*_2_: *p*-value for comparing group I and group III.

*p*_3_: *p*-value for comparing group II and group III.

^*^: Statistically significant at *p* ≤ 0.05.

**Table 2:** Ova stages and ova counts among the studied groups.

**Ova stages**					
**Immature ova**					
Min. – Max.	44.0–59.0	0.0–0.0	20.0–35.0	443.218^*^	<0.001^*^
Mean ± SD	51.0 ± 4.52	0.0 ± 0.0	28.20 ± 4.87		
Median (IQR)	50.50 (48.0–55.0)	0.0	30.0 (24.0– 31.0)		
**Sig. between groups** *p*_1_ < 0.001^*^, *p*_2_ < 0.001^*^, *p*_3_ < 0.001^*^					
**Mature ova**					
Min. – Max.	33.0–49.0	11.0–20.0	19.0–40.0	80.450^*^	<0.001^*^
Mean ± SD	43.20 ± 5.07	14.90 ± 2.33	28.30 ± 6.60		
Median (IQR)	43.50 (40.0– 47.0)	15.0 (14.0– 16.0)	28.0 (25.0– 33.0)		
**Sig. between groups** *p*_1_< 0.001^*^, *p*_2_ < 0.001^*^, *p*_3_ < 0.001^*^					
**Dead ova**					
Min. – Max.	4.0–8.0	76.0–89.0	12.0–55.0	240.564^*^	<0.001^*^
Mean ± SD	5.90 ± 1.29	84.50 ± 3.92	27.0 ± 13.76		
Median (IQR)	6.0 (5.0–7.0)	85.0 (84.0– 87.0)	23.50 (19.0– 30.0)		
**Sig. between groups** *p*_1_ < 0.001^*^, *p*_2_ < 0.001^*^, *p*_3_ < 0.001^*^					
**Ova counts per gram of tissue**					
**Livers**					
Min. – Max.	9,548.0– 19,879.0	1,023.0– 2,145.0	5,325.0– 10,247.0	72.120^*^	<0.001^*^
Mean ± SD	14,066.2 ± 3,747.6	1,312.6 ± 352.6	7,284.9 ± 1,664.0		
Median (IQR)	13,158.0 (11,287.0–16,635.0)	1,164.0 (1,102.0–1,456.0)	6,961.0 (6,187.0–8,262.0)		
**Sig. between groups** *p*_1_ < 0.001^*^, *p*_2_ < 0.001^*^, *p*_3_ < 0.001^*^					
**Intestines**					
Min. – Max.	10,543.0– 14,343.0	1,869.0– 2754.0	6,265.0– 10,321.0	268.654^*^	<0.001^*^
Mean ± SD	12,178.6 ± 1144.8	2,155.6 ± 296.0	8,753.7 ± 1,224.8		
Median (IQR)	12,434.5 (11,236.0–12,553.0)	2,045.5 (1,948.0–2,289.0)	8,908.5 (8,268.0–9,623.0)		
**Sig. between groups** *p*_1_ < 0.001^*^, *p*_2_ < 0.001^*^, *p*_3_ < 0.001^*^					

IQR, Interquartile range; SD, Standard deviation.

F: F for ANOVA test, pairwise comparison between the study groups was done using the *Post Hoc* Test (Tukey).

*p*: *p*-value for comparing the studied groups.

*p*_1_: *p*-value for comparing group I and group II.

*p*_2_: *p*-value for comparing group I and group III.

*p*_3_: *p*-value for comparing group II and group III.

^*^: Statistically significant at *p* ≤ 0.05.

In mice treated with PZQ, the oogram pattern did not reveal any immature ova, while the mean numbers of immature ova were decreased in mice receiving IVM treatment. The mean numbers of mature ova found in treated mice (PZQ and IVM) were reduced. On the other hand, the number of dead eggs in mice treated with PZQ was higher than in mice treated with IVM. Statistically significant differences between (PZQ and IVM) treated mice and the untreated control mice were observed (Table 2).

Table 3 represents the reduction percentages of worm burdens and tissue ova loads. The highest reduction percentage of total worm load, copula, male, and female worms was shown in mice treated with PZQ. All groups' hepatic and intestinal tissues recorded a statistically significant reduction of ova loads. A marked decrease in liver and intestinal ova loads was observed in mice treated with PZQ compared to IVM-treated and control groups.

**Table 3:** Comparison between worm burdens and tissue ova loads reduction percentages.

**Worm burden**	**Reduction percentages**				
	**Non-treated group I (*****n*** = **20)**	**Group II (PZQ) (*****n***= **20)**		**Group III (IV) (*****n*** = **20)**	
	**Mean** ±**SD**	**Mean** ±**SD**	**%**	**Mean** ±**SD**	**%**
Copula	8.9 ± 1.1	0.7 ± 0.4	92	5.4 ± 0.7	39
Males	2.2 ± 0.7	0.8 ± 0.4	64	1.2 ± 0.4	45
Females	0.8 ± 0.7	0.2 ± 0.4	75	0.4 ± 0.5	50
Total worm loads	20.8 ± 1.9	2.4 ± 1.2	88	13 ± 2	37.5
**Tissue ova load**					
Livers	14,066.2 ± 3,441.8	1,312.6 ± 483	90	7,284.9 ± 2,403	48
Intestines	12,178.6 ± 1,061.6	2,155.6 ± 392.7	82	8,753.7 ± 1,011	28

### Histopathological results

**Figure 1:**
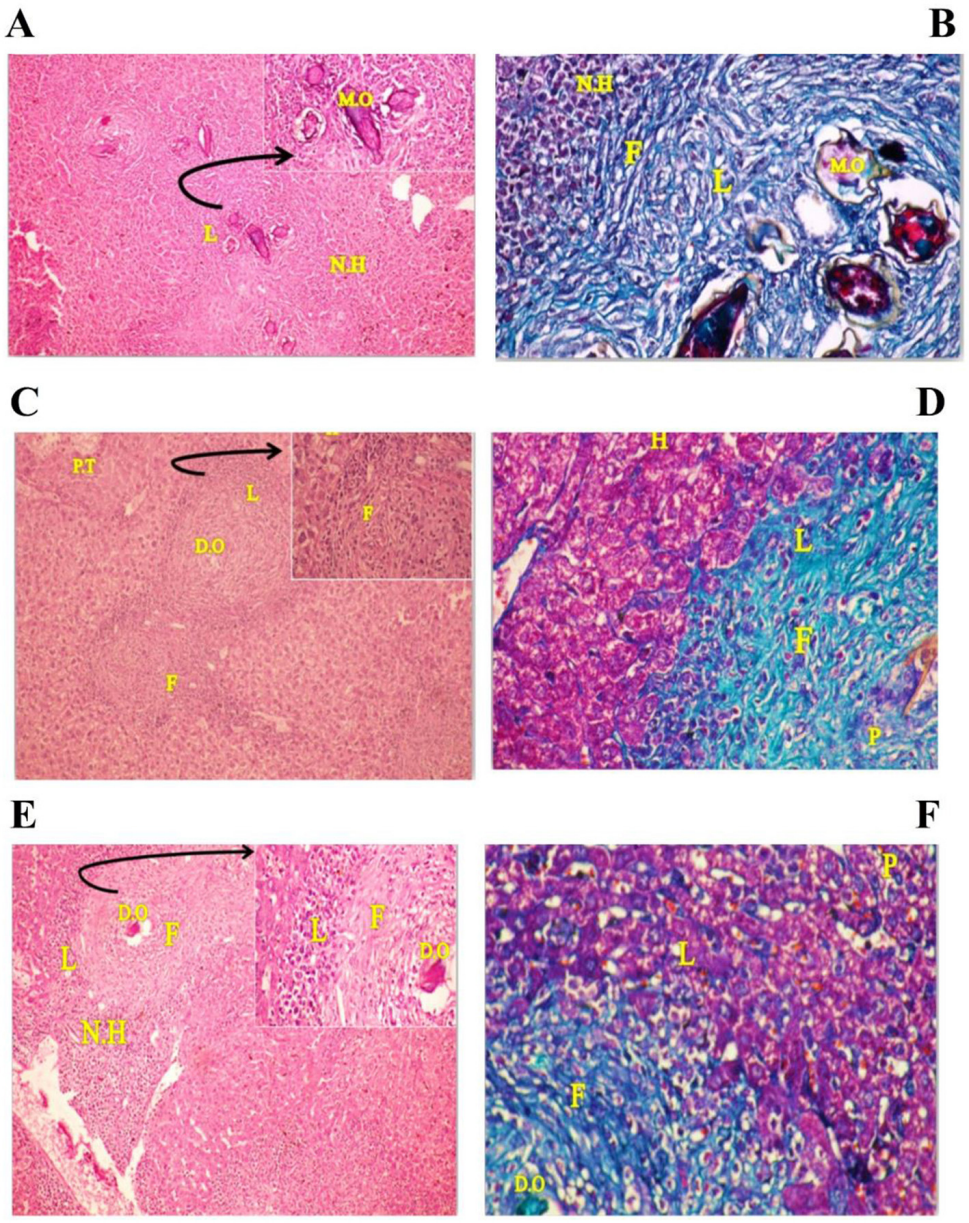
Photographs of stained liver sections of mice. **(A, B)** (group I) showing area of many cellular granulomas surrounded by crowded necrotic hepatocytes (N.H): **(A)** Liver section stained with Hematoxylin-Eosin (H&E × 200) [arrow: high power of the granuloma, note four mature ova (M.O) surrounded by thick area of infiltrating lymphocytes (L)], **(B)** Liver section stained with Masson trichrome (MT × 200) showing thick collagen fibers (F) surrounding the ova as well as internal and external necrotic hepatocytes (N.H) and infiltrating lymphocytes (L). **(C, D)** (group II) showing areas of mild congested portal tract (PT) and central vein (CV) have few infiltrating lymphocytes (L), many recovering hepatocytes (H) have rounded nuclei and homogenous cytoplasm and two cellular granulomas have dead ova (D.O): **(C)** Liver section stained with Hematoxylin-Eosin (H&E × 200) [arrow: high power of the granuloma show infiltrating lymphocytes (L) around many necrotic fibrotic cells (F) surrounded the necrotic lymphocytes (L)], **(D)** Liver section stained with Masson trichrome (MT × 200) showing many pyknotic cells (apoptotic cell) (P), reduction in infiltrating lymphocytes (L), thin collagen fibrotic cells (F) and many recovering hepatocytes (H) at mild congested portal tract region. **(E, F)** (group III) showing areas of many infiltrating lymphocytes (L) near the cellular granulomas surrounded by many necrotic hepatocytes (N.H) and few recovering hepatocytes have rounded nuclei and homogenous cytoplasm: **(E)** Liver section stained with Hematoxylin-Eosin (H&E × 200) [arrow: high power of the granuloma, dead ova (D.O) surrounded by few infiltrating lymphocytes (L) and thick necrotic fibrotic cells (F)], **(F)** Liver sections stained with Masson trichrome (MT × 200) showing area of the granuloma, dead ova (OV) surrounded by few infiltrating lymphocytes (L), mild thick collagen fibrotic cells (F), and many pyknotic one (P) (apoptotic cell).

Untreated mice showed liver parenchyma inflammation, many eosinophilic abscesses, multiple fibrocellular granulomas (70%) around mature ova, and apoptosis in some hepatocytes (Figure 1A). However, PZQ-treated mice showed diminished inflammatory reactions and reduced sizes of granulomata in the liver parenchyma with evident improvement in hepatocytes, the granuloma showed either no ova or degenerated ova with marked reductions in the granuloma numbers and diameters (Figure 1C) and IVM treated mice showed moderate parenchymal inflammation, some eosinophilic abscesses with reductions in granuloma counts and diameters (Figure 1E). Using MT special stain showed that intensive collagen deposition was found within the granuloma of untreated control mice (Figure 1B). In contrast, in PZQ-treated mice (Figure 1D), areas of collagen were diminished, and to a lesser extent in IVM-treated mice (Figure 1F).

### SEM examination results

**Figure 2:**
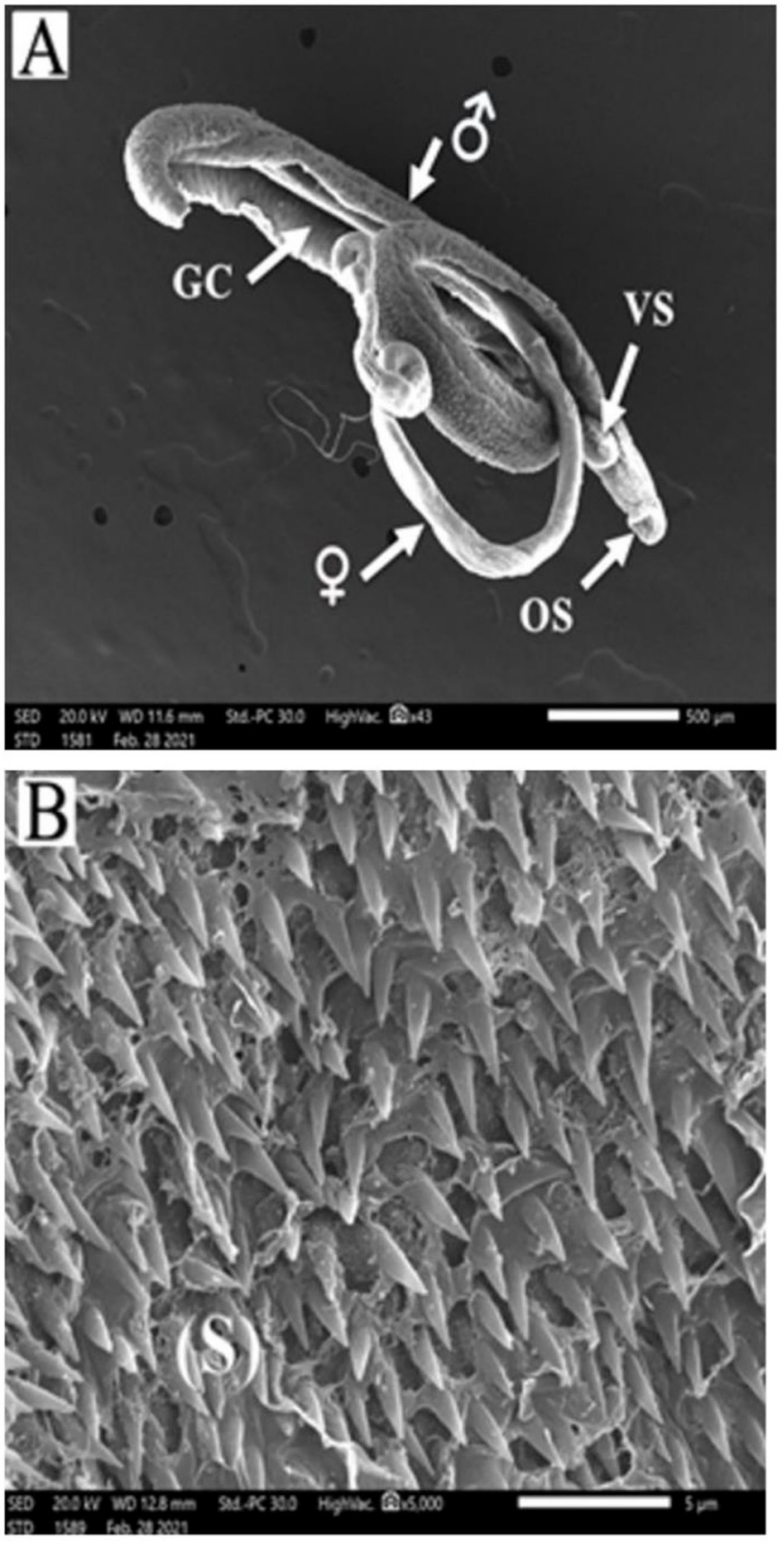
Scanning electron micrographs of *S. mansoni* worms collected from group I control mice. **(A)** Represents a copula with normal tegument, oral sucker (OS), ventral sucker (VS), gynecophoric canal (GC), and male and female worms. **(B)** Showing tegument covered by tubercles with pointed spines (S).

**Figure 3:**
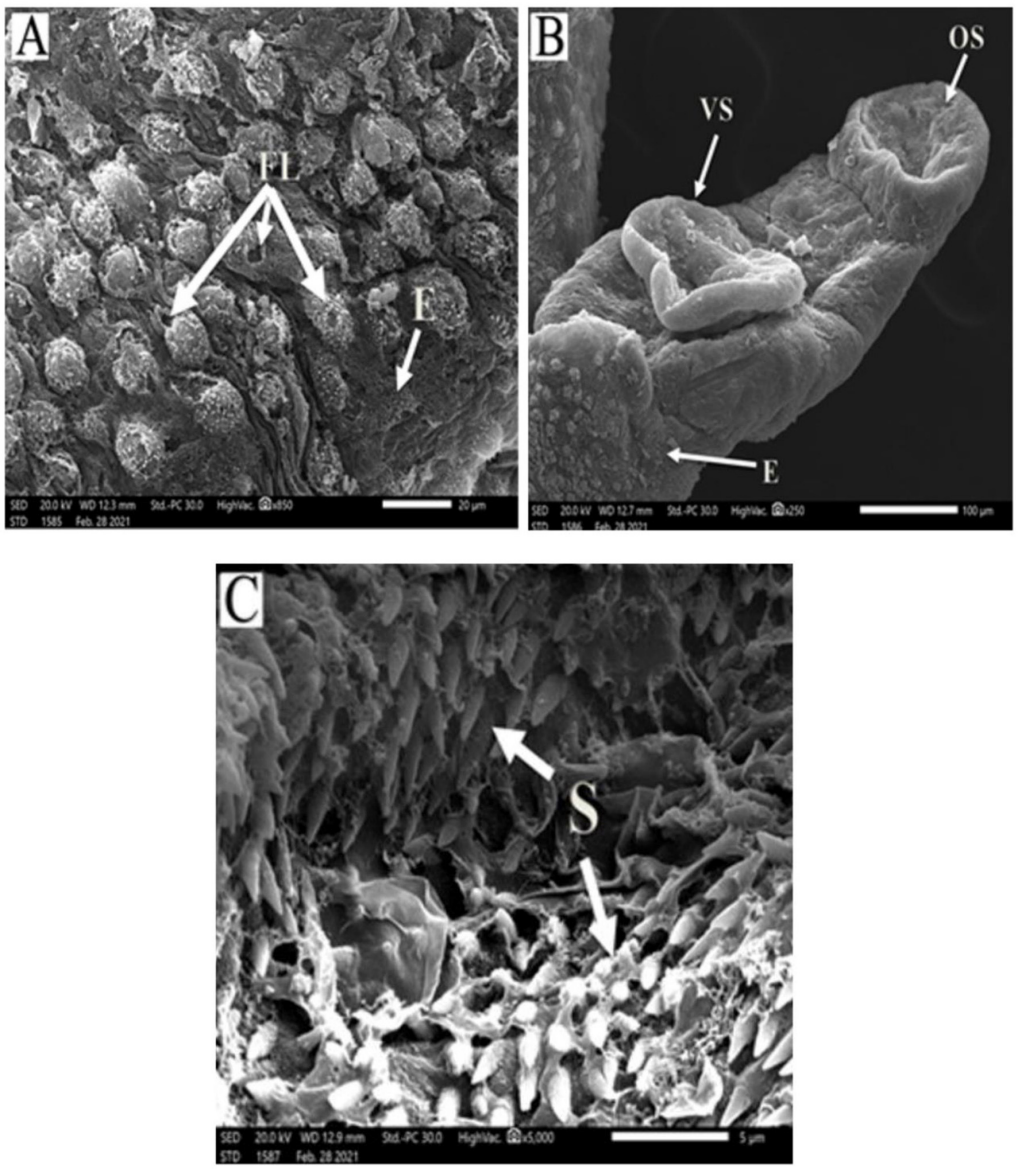
Scanning electron micrographs of *S. mansoni* worms collected from group II PZQ-treated mice. **(A)** Represents tegumental destruction and erosion or loss of tubercles (E), focal lesions (FL), and disappearance of the spines. **(B)** Represents demolish of oral sucker (OS) and ventral sucker (VS), tegument with extensive areas of peeling, and erosion (E) with blebs. **(C)** Showing destruction and loss of tegumental tubercles and spines (S).

**Figure 4:**
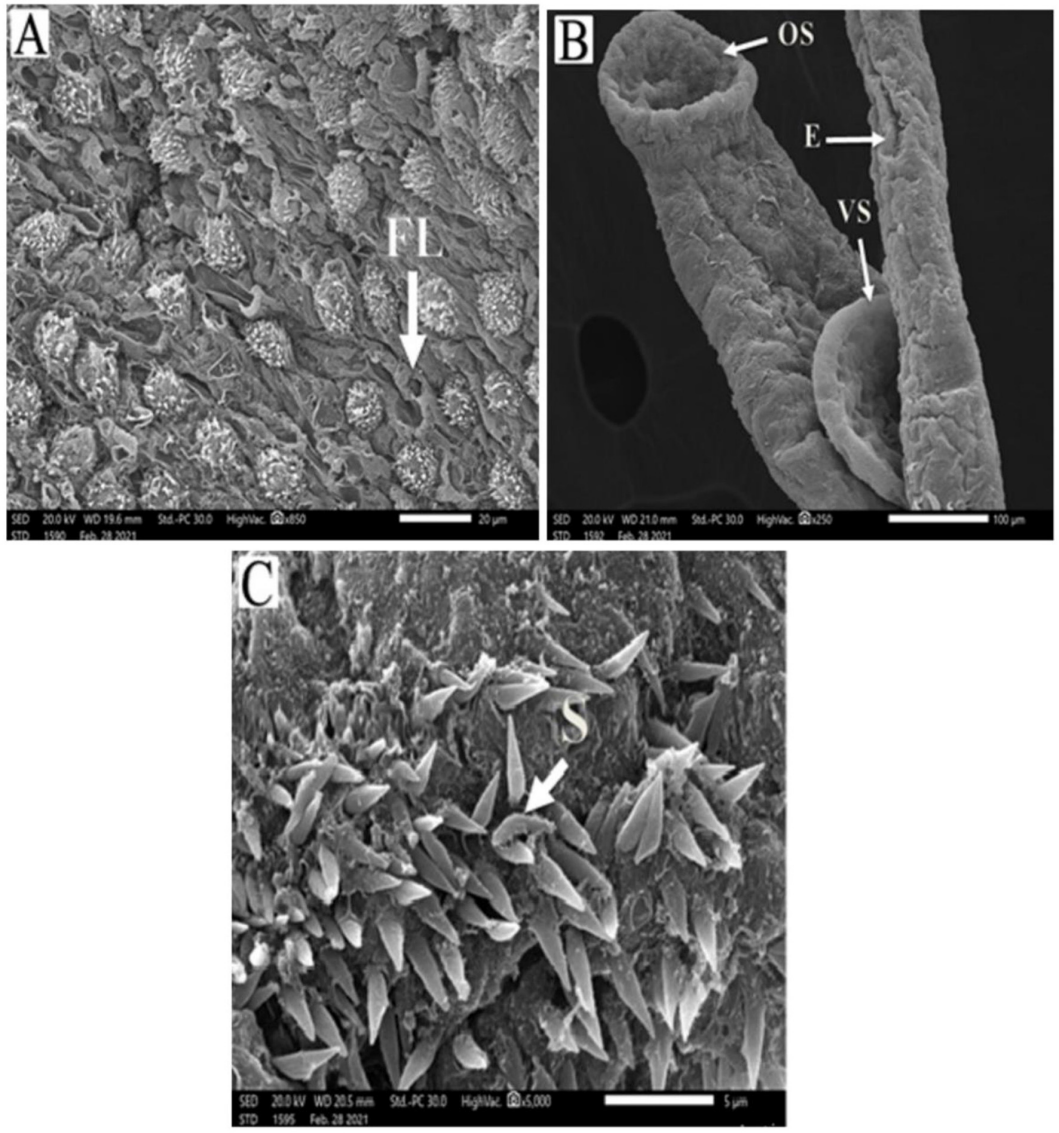
Scanning electron micrographs of *S. mansoni* worm collected from group III IVM-treated mice. **(A)** Represents preferential tegumental destruction, focal lesions (FL), and partial loss of the spines. **(B)** Showed partial damage to oral sucker (OS) and ventral sucker (VS); the tegument showed erosion (E) with blebs. **(C)** Represents destruction and inarranged tegumental spines (S).

*Schistosoma mansoni* male adult worms possess oral and ventral suckers and a pore exhibiting the beginning of the gynaecophoric groove on the ventral surface. In contrast, the dorsal surface has rows of tegumental folds, tegumental ridges, and well-developed tubercles. Figure 2 showed adult worms isolated from the untreated control group showing copula with intact features and organization of oral and ventral suckers, gynecophoric canal (GC) hill-shaped tubercles covered with pointed spines. PZQ-treated mice showed worms with a decreased number of both the tubercles and spines, with areas of focal swellings. Some showed shortening and loss of the tubercles and disappearance of the spines. It also showed noticeable tegumental damage exhibiting sloughing, extensive areas of peeling, severe erosion with blebs, and marked ulceration in the outer surface with various focal lesions. Furthermore, worms showed destruction of the oral and ventral suckers (Figure 3). IVM-treated mice showed worms with less damaging effects on the tegument with localized lesions and fewer tubercles with partial loss of spines. Oral and ventral sucker showed devastation with an erosion of the tegumental layer (Figure 4).

### Proteomic analysis

**Figure 5:**
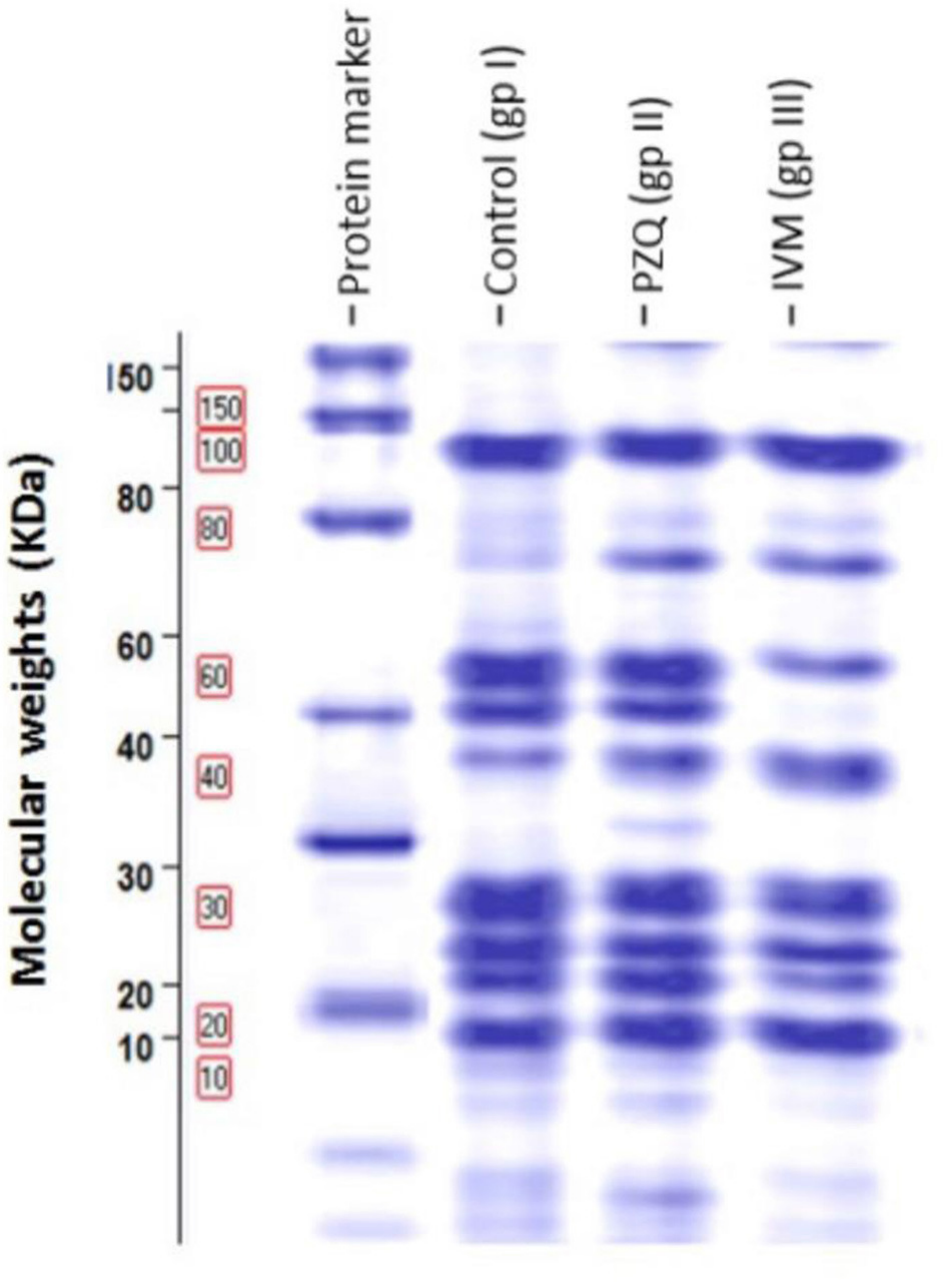
Protein patterns for three samples demonstrating proteins obtained after one-dimensional gel separation by 12% SDS-PAGE and subsequent gel Code Blue staining. Molecular weights in kDa of protein bands were visible on the left of the image of the SDS-PAGE gel.

This study was performed on *S. mansoni* adults harvested from the three studied groups. Differences in protein profile using SDS-PAGE of adult worms treated with PZQ and IVM compared to control were illustrated in Figure 5. Noticeable protein profile changes were recorded as a decrease or increase in protein intensity in each group.

The molecular weights (MWt) of protein bands were analyzed using Total Lab Analysis Software, displaying 19 protein bands distributed in the analyzed worms collected from all groups, as shown in Table 4. The complete disappearance of protein bands number 5 with MWt = 72.79 KDa and band number 17 with MWt = 19.85 KDa in both treated groups was noticed. Furthermore, protein band number 3 disappeared with MWt = 79.96 KDa in PZQ-treated mice rather than those treated with IVM and control mice. A detectable disappearance of protein bands numbers 2, 7, and 16 with MWt = 80.06, 61.59, and 25.75 KDa, respectively, in IVM-treated mice, rather than those treated with PZQ and control-studied mice, was shown in Table 5.

**Table 4:** Variation of protein bands for sample patterns.

**Ref. Band**	**Samples of separated proteins from the worms in the mouse groups**					
	**Control (group I)**		**PZQ (group II)**		**IVM (group III)**	
**Number**	**Band %**	**Molecular weight**	**Band %**	**Molecular weight**	**Band %**	**Molecular weight**
1.	12.02	89.68	11.28	89.68	10.2	89.68
2.	0.34	80.06	3.06	80.06	–	–
3.	1.48	79.96	–	–	1.23	79.96
4.	1.25	79.28	5.11	79.28	6.76	79.28
5.	0.83	72.79	–	–	–	–
6.	9.02	68.74	19.12	68.74	6.42	68.74
7.	9.13	61.59	8.06	61.59	–	–
8.	8.45	54.57	6.57	54.57	4.62	54.57
9.	–	–	3.16	41.57	–	–
10.	4.22	35.49	0.60	35.49	3.42	35.49
11.	12.97	34.81	11.08	34.81	14.69	34.81
12.	11.50	32.24	9.95	32.24	12.83	32.24
13.	9.97	31.35	10.94	31.35	8.13	31.35
14.	11.95	28.94	6.75	28.94	13.63	28.94
15.	0.91	28.05	0.21	28.05	0.64	28.05
16.	1.12	25.75	0.68	25.75	–	–
17.	0.15	19.85	–	–	–	–
18.	1.50	16.04	1.56	16.04	0.35	16.04
19.	1.09	12.06	2.34	12.06	0.67	12.06

**Table 5:** Matrix data of protein bands for sample patterns showing the appearance and disappearance of different protein bands among the studied groups.

**Bands numbers**	**Molecular weights**	**Control group I**	**PZQ treated group II**	**IVM treated group III**
Band 1	89.68	1	1	1
Band 2	80.06	1	1	0
Band 3	79.96	1	0	1
Band 4	79.28	1	1	1
Band 5	72.79	1	0	0
Band 6	68.74	1	1	1
Band 7	61.59	1	1	0
Band 8	54.57	1	1	1
Band 9	41.57	0	1	0
Band 10	35.49	1	1	1
Band 11	34.81	1	1	1
Band 12	32.24	1	1	1
Band 13	31.35	1	1	1
Band 14	28.94	1	1	1
Band 15	28.05	1	1	1
Band 16	25.75	1	1	0
Band 17	19.85	1	0	0
Band 18	16.04	1	1	1
Band 19	12.06	1	1	1

Mass spectrometry (MS) was then used to determine the identity of these bands. As for PZQ-treated mice, the analysis revealed 16 common protein bands shared with the other groups (I and III) with different intensities, while only one protein band (number 9) with Mwt = 41.57 KDa was detected but could not be recognized in the other two groups. Regarding IVM-treated mice, the analysis revealed 13 common protein bands shared with other studied groups (I and II) but with different intensities.

### Mass spectrometry data and bioinformatics analysis

Differential protein bands among the three studied groups were subjected to mass spectrometry to establish their identity. Mascot's search against the NCBInr database with the MS data identified the peptides of *S. mansoni*, which differ between the three groups of all separated samples of SDS-PAGE, representing seven protein bands, as shown in Table 6. Molecular function and biological process were assigned for the potent proteins, as shown in Table 5. Detected protein categories were split into those enzymes associated with the glycolytic pathway, structural proteins, other cytosol stress responses, and chaperones.

**Table 6:** Putative identity of differential band intensities among potent proteins and molecular function of these potent proteins.

**No**.	**Bands no**.	**Potent proteins**		**Groups**		
		**Molecular weights** = **KDa**	**Putative identity/molecular function**	**Control (group I)**	**PZQ (group II)**	**IVM (group III)**
1.	2	80.06	Ca^2+^ ATPase 2 protein/Plasma membrane transporters	**√**	**√**	**–**
2.	3	79.96	Heat shock protein 60/Cytosol stress response and chaperones	**√**	**–**	**√**
3.	5	72.79	Mitogen-activated protein kinase/Cellular responses to stress	**√**	**–**	**–**
4.	7	61.59	Thioredoxin peroxide/Cytosol stress response and chaperones	**√**	**√**	**–**
5.	9	41.57	Heat shock protein 40/Cytosol stress response and chaperones	**–**	**√**	**–**
6.	16	25.75	Tegumental allergen-like protein/Cytoskeleton	**√**	**√**	**–**
7.	17	19.85	Triose phosphate isomerase/Cytosol energy metabolism	**√**	**–**	**–**

## Discussion

Schistosome infection is one of the most significant overall economic and public health burdens and one of the leading causes of helminth-related morbidity and mortality (37). Before eggs' release and entrapment in the liver and gastrointestinal tract tissues on their way to being expelled through the gut, *S. mansoni* infection is relatively insidious. In these organs, eggs trigger strong inflammatory reactions that cause severe granulomatous inflammation and fibrosis (38).

For the treatment of schistosomiasis, PZQ is the only treatment that is easily accessible. The primary intervention for schistosomiasis control programs has been mass PZQ administration due to its low cost, safety, and effectiveness (39). It has been proposed that PZQ damages adult worms irreversibly by interfering with the control of calcium in the parasite membrane. Due to variations in the relevant receptors, mammals are unaffected. Due to the widespread usage of PZQ, resistance is constantly being established. Low cure rates, decreased *S. mansoni* susceptibility to PZQ, and treatment failures have all been documented, which raises questions regarding PZQ resistance and medication effectiveness (40, 41).

IVM, macrocyclic lactone, demonstrates various anthelminthic properties with a documented wide safety margin. Trematodes, nematodes, and arthropods all exhibit the neuronal glutamate-gated chloride channel, which is the target of ivermectin. The versatility of ivermectin makes this drug an interesting compound to be investigated as a medication for other diseases and a potential source for derivatization to improve its activity and efficacy (10).

In the current study, parasitological studies highlighted the potential *in vivo* anti-schistosomal effect of PZQ and IVM drugs with superior effects to PZQ. Regarding worm burden, a statistically significant reduction in the mean copula, males, and total worm loads harvested from PZQ and IVM-treated groups compared with untreated controls was in accordance with (11, 42). On the other hand, (11) recorded a significant reduction in mean female loads, contrary to the present results that showed no statistically significant reduction in mean female worm loads in either PZQ or IVM-treated mice, which is the same as (43). In the PZQ-treated mice, a remarkable reduction of 92%, 64%, 75%, and 88% in copula, males, female worms, and total worm burdens, respectively, was found compared to untreated controls, which are nearly similar to the finding of (44) with a total worm burden reduction of 83% and (45) with a total worm burden reduction of 79%. In the IVM-treated mice, a 39%, 45%, 50%, and 37.5% reduction in copula, males, female worms, and total worm burdens, respectively, were found compared to infected controls. Worm reduction could be attributed to tegumental damage of the worm, which can be verified by SEM, or loss of muscle tone and structures, leading to the death of worms and a decrease in ova laying (46).

Based on the oogram pattern, which assessed the drug effects on the worm oviposition, development, and survival of trapped ova in the intestinal mucosa, the PZQ drug showed a vigorous ovicidal activity as no immature ova (early stage) was found, and reduction in the mature and significant increase in the dead ova recorded in treated mice which led to the overall reduction in tissue ova loads as mentioned in several studies (42, 44, 47). Interestingly, ova from treated mice were smaller and more spherical than those from the control. The reduction in ova size could have a significant effect since smaller ova may become trapped in the body's distal ectopic tissues. However, IVM-treated mice revealed statistically significant differences from controls in the number of immature, mature, and noticeably more dead ova. These findings concur with those of (11) who utilized various IVM doses and found that both showed a significant reduction in immature and mature ova and a significant increase in dead ova.

Regarding tissue ova loads, PZQ and IVM treatments showed a statistically significant reduction in ova load in all treated groups' liver and intestinal tissues. A peerless reduction was demonstrated by PZQ in liver and intestinal tissues with percentages of 90% and 82%, respectively. Moreover, the IVM drug presented a considerable reduction of 48% and 28% in the liver and intestine, respectively. The results of this study mean that decreased tissue ova loads can be considered a hallmark sign of effective anti-schistosomal treatment. In Egypt, (48) documented a significant decrease in the mean number of *S. mansoni* ova per gram of intestine and liver treated with PZQ drug.

Following both treatments, a highly significant decrease in the numbers and sizes of bilharzial granulomas was observed in the liver sections of the mouse groups examined using a routine H&E stain. Furthermore, the MT special stain showed intense collagen deposition within granulomas of the untreated control; by contrast, the areas of collagen were down-regulated after PZQ and to a lesser extent with IVM treatment. These findings agree with those of other studies, which reported a reduction of detected granuloma size, their numbers, and collagen deposition after PZQ administration (45, 47). Several compounds exhibited anti-schistosomal activity by targeting the tegument, such as PZQ and artemether (49). The tegumental changes caused by IVM on adult *schistosomes* recovered from group III mice were also fairly comparable to those seen in other parasites treated with the aforementioned substance as per (11).

In the present work, SEM revealed several morphological changes and damaging effects on adult worms recovered from mice groups treated with PZQ and IVM, which were more extensively affected by PZQ. These ultrastructural alterations are inversely correlated with the effectiveness of PZQ and IV treatment and may help to understand how these worms die. Notably, the lethal effects of PZQ on the adult flukes, as documented through SEM images, were exhibited by injuries and focal lesions upon the surface of schistosomes, which were reported in previous studies (44, 50). Worms appeared shorter than usual due to muscular contraction upon treatment. Furthermore, PZQ destroyed tegument tubercles, which led to the loss of spines that affect the worms' sensory and absorptive functions, which could be another possible mechanism of killing adult worms and reducing ova deposition, decreasing disease. Similar changes, but to a minor extent, were observed in the surface of worms obtained from IVM-treated mice.

Several investigations have documented a synergistic interaction between ivermectin and other medications. A synergistic interaction between remdesivir and ivermectin improved *in vitro* antiviral activity against SARS-CoV-2 (51). In the same way, Hydroxychloroquine and ivermectin displayed a synergistic interaction against COVID-19 (52). The potential advantages of combining IVM with PZQ have been investigated. In MDA programs, the co-administration of PZQ, IVM, and albendazole indicated that such combinations are generally safe (17). Additionally, an IVM/PZQ suspension was developed by (18) with documented improvement of pharmacokinetics in the combination product.

Proteomic analysis of *Schistosoma* proteins has been successfully used in recent years, and it has proven to be a potent method for discovering and identifying proteins specific to the many life stages of the parasite (53–56). In the current study, 19 protein bands were detected; 12 were common between the three studied groups with slightly different intensities, and seven were differentially expressed. Seven selected bands were detected differently in the three groups by SDS-PAGE and subjected to MS to reveal their identities.

The search for PZQ bands against the NCBInr database identified peptides from treated *S. mansoni* as Ca2+ ATPase 2, Thioredoxin peroxidase, Heat shock protein 40, and Tegumental allergen-like protein, which were absent from IVM-treated worms. At the same time, IVM-treated worms possessed heat shock protein 60. Control untreated worms possessed two proteins that were absent from the other two treated groups, which were identified as Mitogen-activated protein kinase and Triose phosphate isomerase. One of the study's most significant findings was the up-regulation of Ca2+ ATPase 2 (Plasma membrane transporters) in *S. mansoni* adult worms treated with PZQ compared to control adult worms, as the same finding was reported by (57).

PZQ may also kill blood flukes by altering protein phosphorylation. The significance of phosphorylation in *S. mansoni* is suggested by detecting differential protein phosphorylation during development using anti-phospho antibodies. Furthermore, the over-expression of Ca2+/calmodulin-dependent protein kinase II (CamKII), a crucial calcium homeostasis kinase observed at sub-lethal PZQ doses, suggests a potential connection between the medication and phosphorylation. Data on how PZQ affects protein phosphorylation in *Schistosoma* parasites might close the knowledge gap about this drug's mode of action and offer hints for future protein phosphorylation-targeting therapeutic development. One significant protein alteration essential to controlling cellular growth, division, and metabolism is phosphorylation (58).

Another remarkable finding of this work was the downregulation of thioredoxin peroxidase (Cytosol stress response and chaperones) detected in PZQ-treated worms compared with control, untreated worms. The current work recorded an inducible expression of heat shock proteins that function as molecular chaperones or proteases, as heat shock protein 60 was expressed in IVM-treated and control groups. In contrast, heat shock protein 40 was detected in PZQ-treated worms only. These findings corroborated those of (59), who reported that heat shock proteins were expressed throughout all stages of the developmental life cycle of *Schistosoma* species. The expression of *S. mansoni* Hsp in the present study is consistent with the findings of (60), who reported that schistosomes showed an increase in heat shock-related mRNA after exposure to PZQ. Furthermore, they showed that PZQ therapy might cause a chemical reaction resembling that seen when schistosomes experience oxidative stress, which clarified the known ineffectiveness of PZQ against juvenile *Schistosoma* worms.

However, at 40 days after infection, schistosomes could no longer respond transcriptionally to drug exposure by producing heat shock proteins, indicating the beginning of PZQ susceptibility against mature worms. Besides the previous proteins, the Tegument-Allergen-Like (TAL) protein is downregulated in PZQ-treated worms. TALs have a significant localization and association with the tegument, a syncytial structure that makes up the organism's outer layer (61, 62). It should be emphasized that another finding in the present study is the detection of Mitogen-activated protein kinases (MAPKs) and Triose phosphate isomerase (TPI) proteins (Cytosol energy metabolism) in the control group and absent from either PZQ or IVM groups.

The identification of differently expressed proteins that could be new pharmacological targets is made possible by proteomic analysis, which offers a thorough insight into the molecular alterations drug treatment brings. Studies have shown that treating *S. mansoni* and *Onchocerca volvulus* with praziquantel (PZQ) and ivermectin (IVM), respectively, changes the expression of proteins involved in critical biological pathways, such as energy metabolism, oxidative stress response, ion transport, and tegument integrity. These proteins could be candidates for new anthelmintic drugs or combination treatments, especially those impacted explicitly by medication exposure (54, 63).

Proteomic signatures can also be used as biomarkers to evaluate the effectiveness of treatments or the emergence of resistance. For example, persistent alterations in the expression of drug transporters or stress response proteins after treatment might point to adaptive reactions that support resistance. Monitoring these genetic markers in field isolates may help identify medication resistance early and enable individualized treatment plans (64).

The current study was limited by the absence of vehicle-only control groups that should be considered in future studies.

## Conclusion

The proteomic analysis detected protein structure changes in the worms pre- and post-treatment that could help pursue drug efficacy on schistosomiasis. This could improve our knowledge about how the drugs used in schistosomiasis treatment work at the molecular level. Praziquantel outperformed ivermectin in terms of parasitological, histopathological, and SEM results; however, the anti-schistosomal properties of ivermectin are encouraging, evident by changes in the protein structure of the worms detected after ivermectin treatment. This may open the way to use ivermectin in combination with other anti-schistosomal medicines to avoid any potential resistance from monotherapy.

We recommend conducting a further comprehensive proteomic analysis using LC/MS-based proteomics to achieve deeper coverage and more precise quantification of differentially expressed proteins. Further functional follow-up validation studies (e.g., qPCR or western blot) are also recommended to support the proteomic findings. Additionally, PZQ and IVM should be combined in schistosomiasis treatment to verify the synergistic effects of both drugs.

## Data Availability

The original contributions presented in the study are included in the article/supplementary material, further inquiries can be directed to the corresponding author.
